# Effects of high-frequency repetitive transcranial magnetic stimulation on the nutritional status of patients in a persistent vegetative state: A pilot study

**DOI:** 10.3389/fnut.2023.924260

**Published:** 2023-03-23

**Authors:** Xuan-Wei Liu, Na-Na Zhao, Tao Pang, Qiong Wen, Peng Xiao, Ke-Xue Zeng, Dan-Ning Wang, Jia-Min Chen, Yu-Long Wang, Hai-Bo Yu

**Affiliations:** ^1^Department of Rehabilitation, Shenzhen Dapeng New District Nan’ao People's Hospital, Shenzhen, China; ^2^Department of Acupuncture and Massage, Shenzhen Luohu District Hospital of Chinese Medicine, Shenzhen, China; ^3^Department of Rehabilitation, Guangdong Province Second Hospital of Chinese Medicine, Guangzhou, China; ^4^Department of Rehabilitation, Shenzhen Second People's Hospital, Shenzhen, China; ^5^Department of Acupuncture and Massage, The Fourth Clinical Medical College of Guangzhou University of Chinese Medicine, Shenzhen, China

**Keywords:** high-frequency repetitive transcranial magnetic stimulation, persistent vegetative state, nutritional state, rehabilitation, acupuncture and massage

## Abstract

**Purpose:**

This paper presents a preliminary study on whether repetitive transcranial magnetic stimulation (rTMS) can modulate the nutritional status of persistent vegetative state (PVS) patients (the primary endpoint) by regulating the intestinal flora and the metabolites, with the correlation between them also investigated.

**Methods:**

Seventy-six patients with PVS were selected and divided into the observation group (*n* = 38) and the control group (*n* = 38) by random numerical grouping. All subjects’ stool samples were examined for metabolites and analyzed regarding the short-chain fatty acids (SCFAs) content. All subjects’ serum albumin, prealbumin, and hemoglobin levels were measured before and after the treatment. Nutrition risk screening 2002 was performed on all the subjects before and after the treatment and on the 30th and 90th days of the follow-up.

**Results:**

(1) Intestinal flora structure: the Chao index, Ace index, and Shannon index of the observation group and the control group were significantly higher (*p* < 0.05), while the Simpson index was significantly lower (*p* < 0.05) following the treatment. (2) Metabolites of the intestinal flora: the observation group had significantly higher levels of acetic acid, butyric acid, and valeric acid (*p* < 0.05), as well as lower levels of propionic acid (*p* < 0.05) following the treatment. (3) Nutritional status (the primary endpoint): following the treatment, the above serum nutritional indices were significantly higher in both groups (*p* < 0.05), while the indices of the observation group were significantly higher than those of the control group (*p* < 0.05).

**Conclusion:**

The rTMS method may improve the nutritional status of patients with PVS by regulating the structure of the intestinal flora and affecting the level of SCFAs through the microbiota–gut–brain axis. The possible mechanism involves how high-frequency rTMS can cause increased excitation in the frontal lobe of the right side of the brain, thus regulating the 5-hydroxytryptamine and norepinephrine levels.

## 1. Introduction

A persistent vegetative state (PVS) is a state of vegetation lasting more than 1 month due to severe brain injury. PVS patients experience a complete loss of their cognitive functioning in relation to themselves and the outside world. However, they retain certain autonomic functions, albeit without spontaneous speech or purposeful movements of their extremities (1). Furthermore, such patients tend to experience subcortical unconscious activity and may exhibit painful expressions or avoidance responses to painful or noxious stimuli, although they generally exhibit no orienting responses. They may also cry unconsciously or have an irregular sleep–wake cycle.

With the advancement of technology, the development of intensive care equipment, and the improvement of emergency treatment technology, the mortality rate of emergency patients has been significantly reduced; however, many patients are still experiencing a PVS (2). According to the statistics, the global incidence of PVS patients is around 0.03% (3), and 0.19% in the United States (4), while it is estimated that there are approximately 150,000 PVS patients in China. Meanwhile, the disease is marked by poor prognosis, poor recovery of consciousness and functioning, and high rates of disability and mortality. Poor nutrition can also aggravate the primary disease, increase the attendant complications, prolong the hospitalization period, increase the related costs, and increase the mortality rates (5). A retrospective study in China found that the mortality risk in PVS patients with poor nutritional status was 3.352× higher than in patients with good nutritional status (6). It was also found that sustained enteral nutrition therapy could improve the nutritional status of PVS patients and, subsequently, the prognosis of the disease too. The intestinal microbiota is a large community of microorganisms that constitutes the intestinal microecosystem of the body and directly influences the body’s relative health. The intestinal tract provides a habitat for microbiota and the nutrients they need, down-regulates the immune responses to develop immune tolerance, and promotes their colonization in the intestinal tract. The complex interactions among the gastrointestinal hormones, microbial-derived metabolites, and energy homeostasis are considered to be part of the brain–gut axis. Therefore, it can be stated that the microbiota–gut–brain (MGB) axis is a bidirectional response system that combines the host’s gut and brain activities and provides a potential pathway for the intestinal flora and its metabolites to enter the brain.

Repetitive transcranial magnetic stimulation (rTMS) is a physical neuromodulation technique that incorporates a pulsed magnetic field to act on the central nervous system (mainly the brain) in view of altering the membrane potential of cortical nerve cells, causing them to generate induced currents that affect the metabolism and the neuroelectrical activity in the brain, thereby causing a series of physiological and biochemical reactions (7).

The bidirectional information exchange system between the intestine and the brain is known as the gut–brain axis. In recent years, an increasing number of studies have demonstrated that intestinal flora can regulate the development and functioning of the brain through the gut–brain axis, which, in turn, affects the host’s behavior. Following this, medical scientists have proposed the concept of the MGB axis (8). The communication within the MGB axis indicates how signals from the intestinal flora affect brain functioning and how the brain influences intestinal flora’s activity and the gastrointestinal tract’s physiology. Such two-way communication is achieved mainly through the neuroendocrine and neuroimmune mechanisms of the autonomic nervous system and the enteric nervous system. The MGB axis communication pathways include the involvement of the autonomic nervous system (ANS) (e.g., biogenic amines: epinephrine, norepinephrine). Exogenous nerves stimulate efferent nerve fibers, directly or indirectly affecting local intestinal function, that is, the movement, secretion, and thus the composition of the microbiome (9). One recent preclinical mouse study highlights the ability of rTMS to modulate monoamine transporter expression and increase messenger ribonucleic acid levels of dopamine and norepinephrine transporters (DATs and NETs). In the latter case, the increased uptake and binding of norepinephrine in the mouse brain resulted in the reduced bioavailability of norepinephrine (10). Norepinephrine is reported to stimulate the growth of various intestinal pathogens and increase the toxicity of other pathogens such as *Campylobacter jejuni* or *Escherichia coli* (11).

With this in mind, guided by the concept of the bidirectional regulation of the MGB axis, the primary endpoint of this study is the effect of rTMS treatment on the nutritional status of patients in a PVS through observing the treatment of patients in a PVS using this technique.

## 2. The study sample

Seventy-six PVS patients who received treatment in the vegetative consciousness-promoting unit of our hospital from December 2017 to April 2020 were selected. Among them, 47 were men, and 29 were women; they were aged from 18 to 80, with a mean age of 48.32 ± 7.57 years. The 76 PVS patients were randomly divided into the observation group (*n* = 38) and the control group (*n* = 38) using the random number grouping method along with Excel. Regarding the comparison of the general data between the two groups, there was no statistically significant difference (*p* > 0.05) in terms of age (*p* = 0.810), disease course (*p* = 0.171), and gender (*p* = 0.813). The etiologies of the two groups included cerebral hemorrhage, traumatic brain injury, carbon dioxide poisoning, and hypoxic encephalopathy (*p* = 0.431), with the differences not statistically significant (*p* > 0.05). This study was approved by the Ethics Committee of Shenzhen Dapeng New District Nan’ao People’s Hospital (200812114531659–63).

For inclusion in this study, the patients had to meet the following criteria:

1. Had met the diagnostic criteria for PVS.
2. Were aged between 18 and 80 years.
3. Were willing to be treated with nutritional support *via* only enteral nutrition or homemade homogenized formulae according to the requirements of this study.
4. Had a body mass index of between 18.5 and 23.9.
5. Had family members or authorized delegates who agreed to participate in this study and had signed the informed consent form.

Meanwhile, the criteria for exclusion were as follows:

1. Patients that were treated with antibiotics and probiotic preparations within 2 weeks.
2. Patients experiencing complications pertaining to severe endocrine and metabolic diseases.
3. Patients experiencing complications pertaining to severe hepatic and renal dysfunction.
4. Patients experiencing complications pertaining to severe cardiopulmonary insufficiency.
5. Patients experiencing complications due to peptic ulcers, bleeding, and severe diarrhea, which prevented the application of enteral nutrition preparations.
6. Patients experiencing complications pertaining to rheumatic immune diseases.
7. Patients experiencing complications due to malignant tumors.
8. Any conditions with no access to high magnetic field conditions (cardiac pacemakers, deep brain stimulators, vagus nerve stimulators, metal internal fixation appliances, etc.).
9. Patients had a previous history of epilepsy.
10. Patients experiencing organic brain disorders.

## 3. Therapeutic plans

### 3.1. Therapeutic methods used for the observation group

#### 3.1.1. Conventional treatment

This includes treatment of the underlying disease and pharmaceutical treatment to promote consciousness. The treatment of the underlying disease includes the use of antihypertensive, hypoglycemic, antiplatelet coagulation, anticoagulation, and statins, while the consciousness-promoting treatment includes the use of drugs such as bromocriptine, dobutamine, and amantadine.

#### 3.1.2. Rehabilitation training

This includes exercise therapy and swallowing function training (both passive), medium-frequency pulsed electric therapy, and pneumatic therapy.

#### 3.1.3. Nutritional treatment

This includes taking enteral nutritional suspension (TPF) containing ω-3 polyunsaturated fatty acids (TPF, Nutrison MF, Nutricia Pharmaceutic Co., Ltd.), each 500 kcalvsolution contains: Protein 20 g, Fat 19.5 g, Carbohydrate 61.5 g, dietary Fiber 7.5 g, Minerals 2.5 g, Vits 150 mg. This solution is suitable for patients who have gastrointestinal function or partial gastrointestinal function and cannot or are unwilling to eat enough amount of regular food to meet the needs of the body for enteral nutrition treatment. With the provision of 25–30 kcal/kg per day.

#### 3.1.4. High-frequency repetitive transcranial magnetic stimulation

This form of treatment was performed based on conventional treatment and rehabilitation therapy. A CCY-1 TMS instrument (Wuhan Yiruide Group) was used, with the treatment probe a coil shaped like a number “8.” The PVS patients were placed in a lying position prior to the treatment. The magnetic stimulation coil was placed at the center of the coil through the positioning cap at the target stimulation point and tangential to the skull surface, with the target stimulation point located in the right dorsolateral prefrontal cortex area. The frequency was 15 Hz, and the magnetic stimulation intensity was 80–120% of the movement threshold (depending on the different movement thresholds of the individual patients). The course of each stimulation treatment was 6 s with an interval of 28 s, while the stimulation volume was 1,050 pulses. There was one 20 min stimulation treatment each day. The treatment was carried out continuously for 6 days per week with an interval of 1 day for a total of 4 weeks.

### 3.2. Therapeutic methods used for the control group

This included conventional treatment alongside rehabilitation therapy and nutrition therapy, all of which were the same as those used for the observation group. The course of the treatment was 4 weeks.

## 4. Observation indices

1. Intestinal flora and metabolite detection: Stool samples were collected from all the subjects 1–2 day before the treatment and 1–2 day after the treatment (some patients could not defecate once a day), with deoxyribonucleic acid extracted from the intestinal flora of the samples.
2. Polymerase chain reaction amplification of the 16S ribosomal RNA gene V3–V4 region and sequencing of the MiSeq library: The sequencing was performed using the double-ended PE300 strategy according to the instrument’s manual, while the image analysis and data conversion process were performed using the software included in the instrument.
3. Gene data analysis of the intestinal flora: Alpha diversity indices, including the Chao index, Ace index, Shannon index, and Simpson index, were calculated using the QIIME software package.
4. Quantitative determination of metabolites in the intestinal flora: The stool metabolites were quantitatively measured using high-performance liquid chromatography-mass spectrometry.
5. Evaluation of the nutritional state: The immunoblotting test measured the patients’ serum albumin and prealbumin levels, and their hemoglobin level was measured by colorimetry before and after treatment. All the subjects were evaluated using the Nutrition Risk Screening Scale (NRS-2002, a keynote presentation of the European Society for Parenteral Enteral Nutrition [ESPEN] at its annual meeting in Munich, Germany, 2002, published in the Journal of Clinical Nutrition in 2003, as recommended by ESPEN guidelines) (12) before and after the treatment and on the 30th and 90th days of the follow-up. The PVS patients could not be weighed and were considered to be at risk of malnutrition with fasting serum albumin of less than 30 g/l in the morning (three points or greater equates to nutritional risk).

## 5. Statistical methods

**Figure 1:**
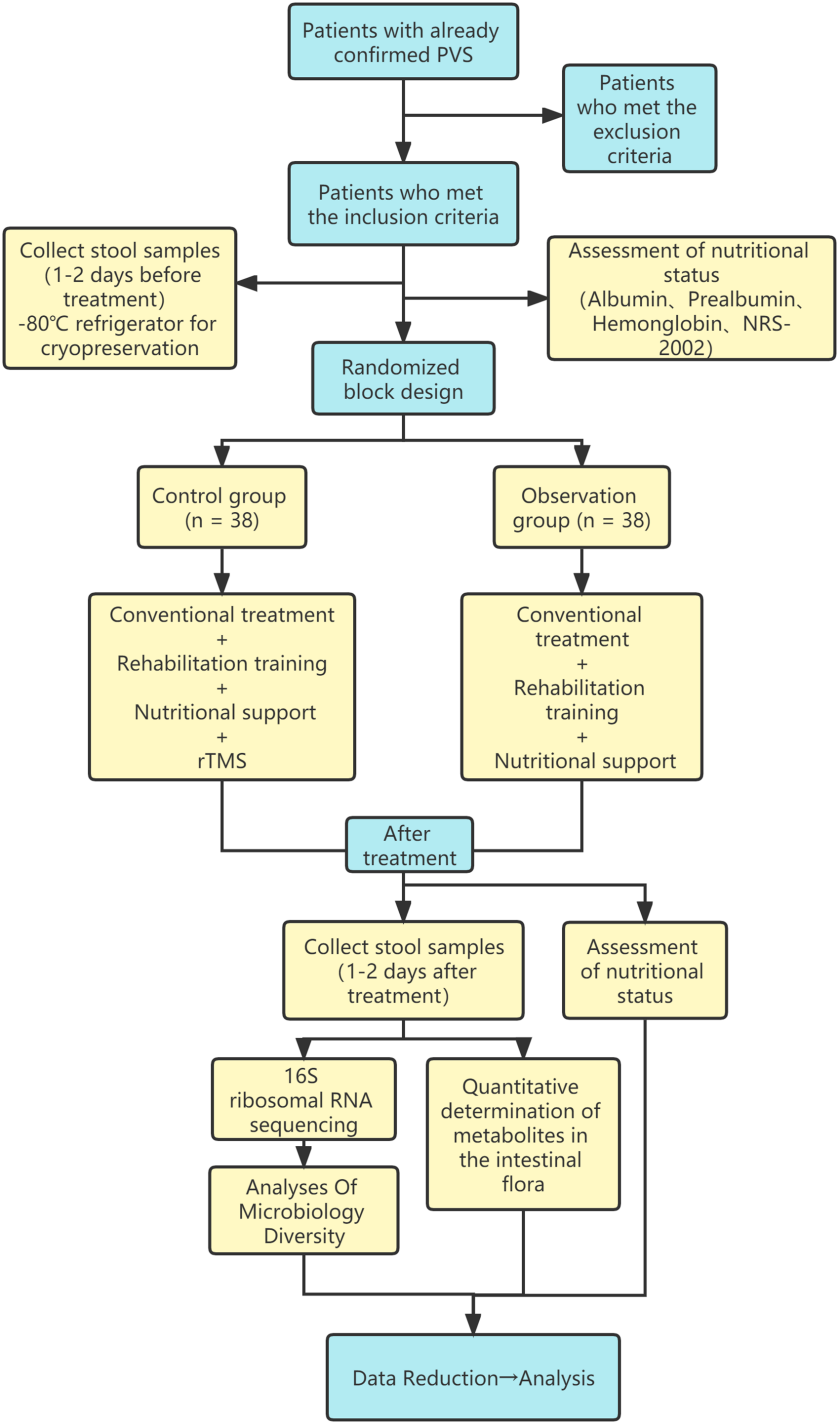
The flow chart of this experiment.

The SPSS 22.0 program was used for the statistical processing, with the data expressed as mean ± standard deviation ( $\bar{x}\pm s$ ), while the repeated-measures method was used to analyze the place navigation results. The remainder of the experiments were compared pairwise using the one-way analysis of variance and Student–Newman–Keuls methods. The normally distributed data with even variance were compared pairwise using a *t*-test, while the data with non-normal distribution and/or uneven variance were expressed as median (quartiles) and compared pairwise using a rank-sum test (the Kruskal–Wallis method) and multi-group ranking. Differences of *p* < 0.05 were regarded to be statistically significant. The flow chart of this experiment see Figure 1.

## 6. Results

Overall, it was found that the rTMS treatment had a positive effect on the prognosis of the patients. See data summary in Tables 1–5.

**Table 1:** The data before and after the TMS treatment of the different studied variables (Intestinal flora).

Flora	Control group (*n* = 38)		*t*	*p*	Treatment group(*n* = 38)		*t*	*p*
	Before (%)	After (%)			Before (%)	After (%)		
*Bacteroides*	9.31 ± 1.27	13.25 ± 2.01	10.215	0.000	9.21 ± 1.34	10.97 ± 2.66^a^	3.643	0.001
*Prevotella*	0.35 ± 0.09	0.54 ± 0.08	9.723	0.000	0.32 ± 0.10	0.41 ± 0.11^a^	2.903	0.005
*Faecalibacterium*	10.71 ± 2.04	14.22 ± 3.12	5.804	0.000	10.69 ± 2.16	12.17 ± 2.45^a^	2.793	0.007
*Faecalibacterium*	0.46 ± 0.10	0.56 ± 0.11	4.147	0.000	0.46 ± 0.09	0.51 ± 0.07^a^	2.291	0.025
*Enterobacteriaceae*	0.47 ± 0.09	0.40 ± 0.04	4.381	0.000	0.47 ± 0.10	0.43 ± 0.05^a^	2.205	0.031
*Pseudomonodaceae*	0.19 ± 0.08	0.11 ± 0.07	4.639	0.000	0.19 ± 0.07	0.15 ± 0.08^a^	2.320	0.023
*Ralstonia*	0.16 ± 0.09	0.07 ± 0.05	5.389	0.000	0.16 ± 0.05	0.11 ± 0.08^a^	3.267	0.002

**Table 2:** The data before and after the TMS treatment of the different studied variables (Stool metabolites).

	*N*	Acetic acid		Propionic acid		Butyric acid		Pentanoic acid	
		Before (%)	After (%)	Before (%)	After (%)	Before (%)	After (%)	Before (%)	After (%)
Treatment group	38	29.31 ± 5.27	35.95 ± 6.01^a^	16.45 ± 1.34	10.27 ± 3.66^a^	19.21 ± 2.34	23.97 ± 2.66^a^	7.09 ± 1.22	9.37 ± 2.61^a^
Control group	38	29.35 ± 6.09	31.54 ± 6.08	16.32 ± 1.10	15.41 ± 4.11	19.23 ± 2.12	20.01 ± 2.11	7.02 ± 1.19	7.62 ± 2.03
*T*		*t* = 0.030	*t* = 3.116	*t* = 0.452	*t* = 5.467	*t* = 0.038	*t* = 7.035	*t* = 0.248	*t* = 3.192
*p*		0.976	0.003	0.652	0.000	0.970	0.000	0.805	0.002

**Table 3:** The data before and after the TMS treatment of the different studied variables (Serum Nutritional Indexes).

Group	*N*	Albumin (g/L)		Prealbumin (mg/L)		Hemoglobin (g/L)	
		Before	c	Before	After	Before	After
Treatment group	38	27.52 ± 5.14	34.31 ± 4.59^a^	90.23 ± 17.45	169.14 ± 20.48^a^	101.23 ± 21.14	134.13 ± 16.95^a^
Control group	38	28.01 ± 5.22	31.25 ± 7.13^a^	90.96 ± 16.74	135.48 ± 25.73^a^	100.59 ± 20.99	119.65 ± 17.08^a^
*T*		*t* = 0.404	*t* = 2.186	*t* = 0.182	*t* = 6.193	*t* = 0.130	*t* = 3.635
*P*		0.687	0.032	0.856	0.000	0.897	0.001

**Table 4:** The data before and after the TMS treatment of the different studied variables (NRS2002).

Group	*N*	Before	After	30 day after treatment	90 day after treatment
Treatment group	38	5.03 ± 1.14	4.13 ± 0.95^a^	3.63 ± 0.69^ab^	3.02 ± 1.01^abc^
Control group	38	4.99 ± 1.21	4.64 ± 0.98	4.16 ± 0.87^ab^	3.59 ± 1.21^abc^
*T*		*t* = 0.145	*t* = 2.258	*t* = 2.888	*t* = 2.187
*P*		0.885	0.027	0.005	0.032

**Table 5:** The variations at the time-points.

Relevant Factor	rTMS group (*n* = 38)		*t*	*p*	Control group (*n* = 38)		*t*	*p*
	Before treatment	After treatment			Before treatment	After treatment		
Weight (kg)	63.99 ± 15.56	65.64 ± 17.82	0.429	0.669	67.76 ± 15.45	68.228 ± 14.97	0.149	0.882
BMI (kg/cm^2^)	23.06 ± 4.84	21.15 ± 3.27	1.994	0.047	22.56 ± 5.84	22.98 ± 6.07	0.307	0.759
MAC (cm)	22.97 ± 4.08	23.24 ± 5.01	0.258	0.797	23.11 ± 5.21	23.76 ± 5.79	0.514	0.609
CC (cm)	27.60 ± 5.82	28.37 ± 6.42	0.548	0.5855	27.26 ± 5.26	28.37 ± 5.86	0.478	0.634
AMC (cm)	20.24 ± 3.94	22.48 ± 4.57	2.289	0.025	20.67 ± 3.48	21.26 ± 4.03	0.683	0.497
TSF (mm)	7.70 ± 2.55	8.1 ± 3.03	0.622	0.535	8.94 ± 4.04	9.13 ± 4.25	0.199	0.842

MAC, Upper Arm Circumference; CC, Calf Circumference; AMC, Arm muscle Circumference; TSF, Triceps skin-fold.

### 6.1. The MiSeq sequencing results

The coverage index of the observation group was 0.9985 ± 0.0022, while that of the control group was 0.9979 ± 0.0019. The coverage index of both groups was greater than 0.99, indicating that the probability of a sequence not being detected in the samples was low and that the sequencing results could reflect the real situation of the stool samples.

### 6.2. Alpha diversity analysis of the intestinal flora

**Figure 2:**
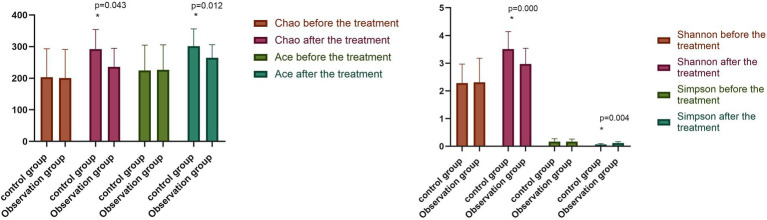
Comparison of intestinal flora diversity between two groups before and after the treatment (^−^*x* ± *s*). Compared with the control group after the treatment, *p < 0.05.

The differences between the alpha diversity of the two groups before the treatment were not statistically significant (*p* > 0.05). Conversely, following the treatment, the Chao index, Ace index, and Shannon index of the observation group and the control group were significantly higher (*p* < 0.05), with the Simpson index significantly lower (*p* < 0.05) for both groups compared with beforehand. Meanwhile, the Chao index, Ace index, and Shannon index of the stool flora of the observation group were significantly higher than those of the control group (*p* < 0.05), while the Simpson index of the observation group was significantly lower than that of the control group (*p* < 0.05), as shown in Figure 2.

### 6.3. Changes in intestinal flora

**Figure 3:**
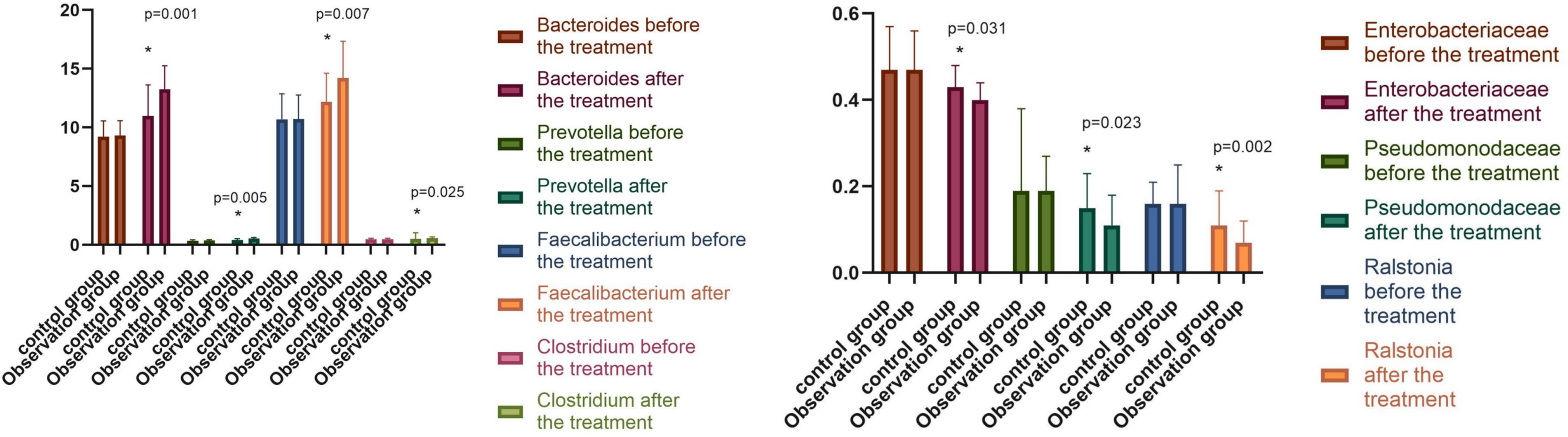
Changes in intestinal flora in both groups before and after the treatment (^−^*x* ± *s*). Compared with the control group after the treatment, *p < 0.05.

The relative abundance of *Bacteroides, Prevotella, and Faecalibacterium* in the two groups before the treatment was compared, with the difference found to be not statistically significant (*p* > 0.05). The relative abundance of *Bacteroides, Prevotella, Faecalibacterium,* and *Clostridium* in the observation group increased significantly (*p* < 0.05) after the treatment compared with that before. In contrast, the relative abundance of *Enterobacteriaceae, Pseudomonodaceae,* and *Ralstonia* was significantly reduced (*p* < 0.05). The relative abundance of *Bacteroides, Prevotella, Faecalibacterium,* and *Clostridium* in the control group was significantly higher (*p* < 0.05) following the treatment compared with that before the treatment. Conversely, the relative abundance of *Enterobacteriaceae, Pseudomonodaceae,* and *Ralstonia* was significantly reduced (*p* < 0.05). Following the treatment, the differences in the relative abundance of intestinal flora in the observation group were statistically significant (*p* < 0.05), as shown in Figure 3.

### 6.4. Metabolites of the intestinal flora

**Figure 4:**
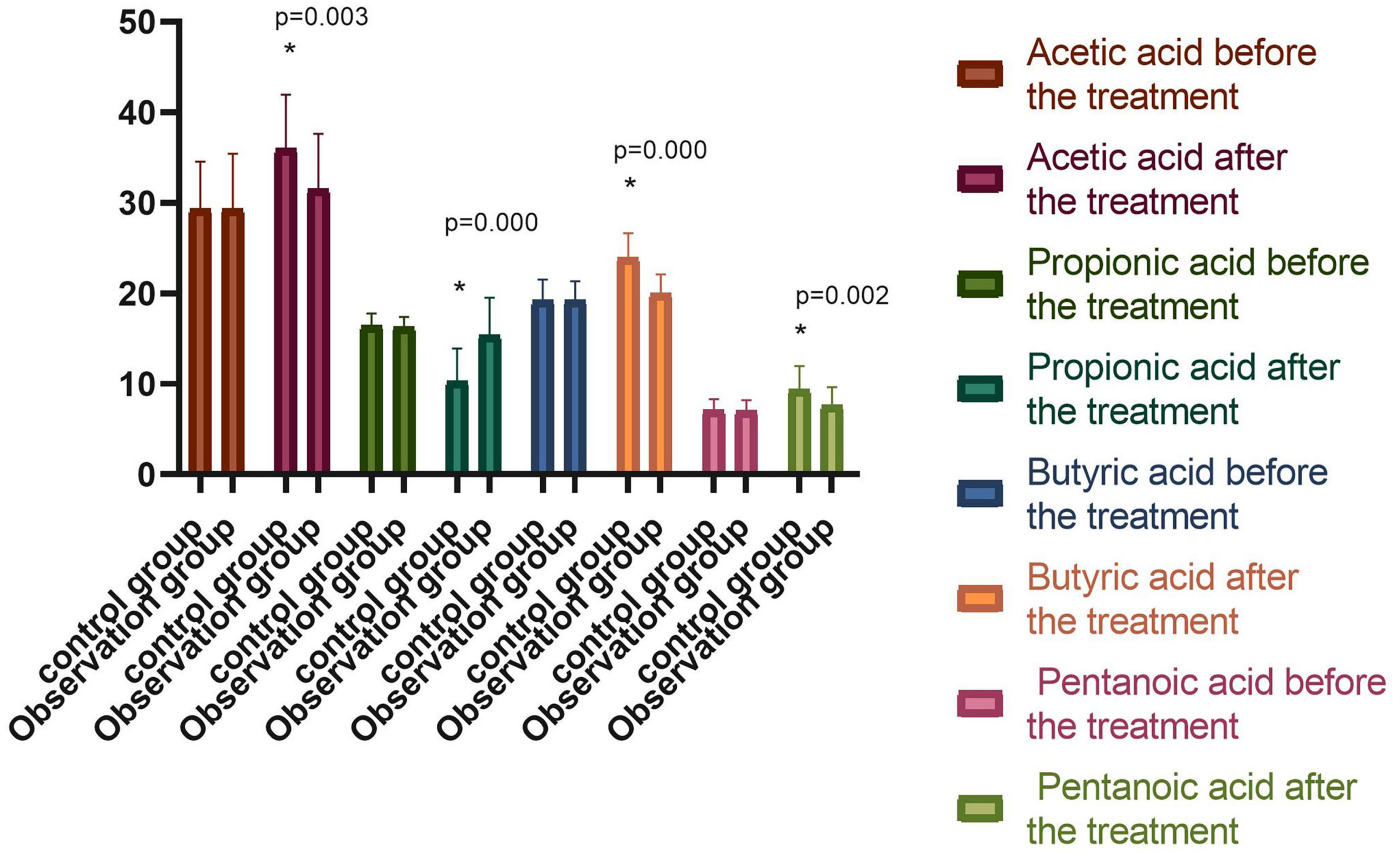
Comparison of the percentage of peak areas of metabolites of intestinal flora between the two groups before and after the treatment (^−^*x* ± *s*). Compared with the group before the treatment, *p < 0.05.

The short-chain fatty acids (SCFAs) in the stool samples were mainly acetic acid, propionic acid, butyric acid, and valeric acid, which were the metabolites of the intestinal flora in the two groups before the treatment. When comparing the peak areas of the four compounds, it was found that the acetic acid had the highest content, and the valeric acid had the lowest. The differences were not statistically significant (*p* > 0.05) in the comparison of acetic acid, propionic acid, butyric acid, and valeric acid. Following the treatment, the levels of acetic acid, butyric acid, and valeric acid in the observation group were significantly higher (*p* < 0.05), while the level of propionic acid was significantly lower (*p* < 0.05) compared with beforehand. Meanwhile, the control group exhibited an increase in acetic acid, butyric acid, and valeric acid, and a reduction in propionic acid following the treatment, while the difference was not statistically significant (*p* > 0.05). The difference in the metabolites of the intestinal flora of the observation group was statistically significant compared with the observation group following the treatment (*p* < 0.05), as shown in Figure 4.

### 6.5. Nutritional evaluation

**Figure 5:**
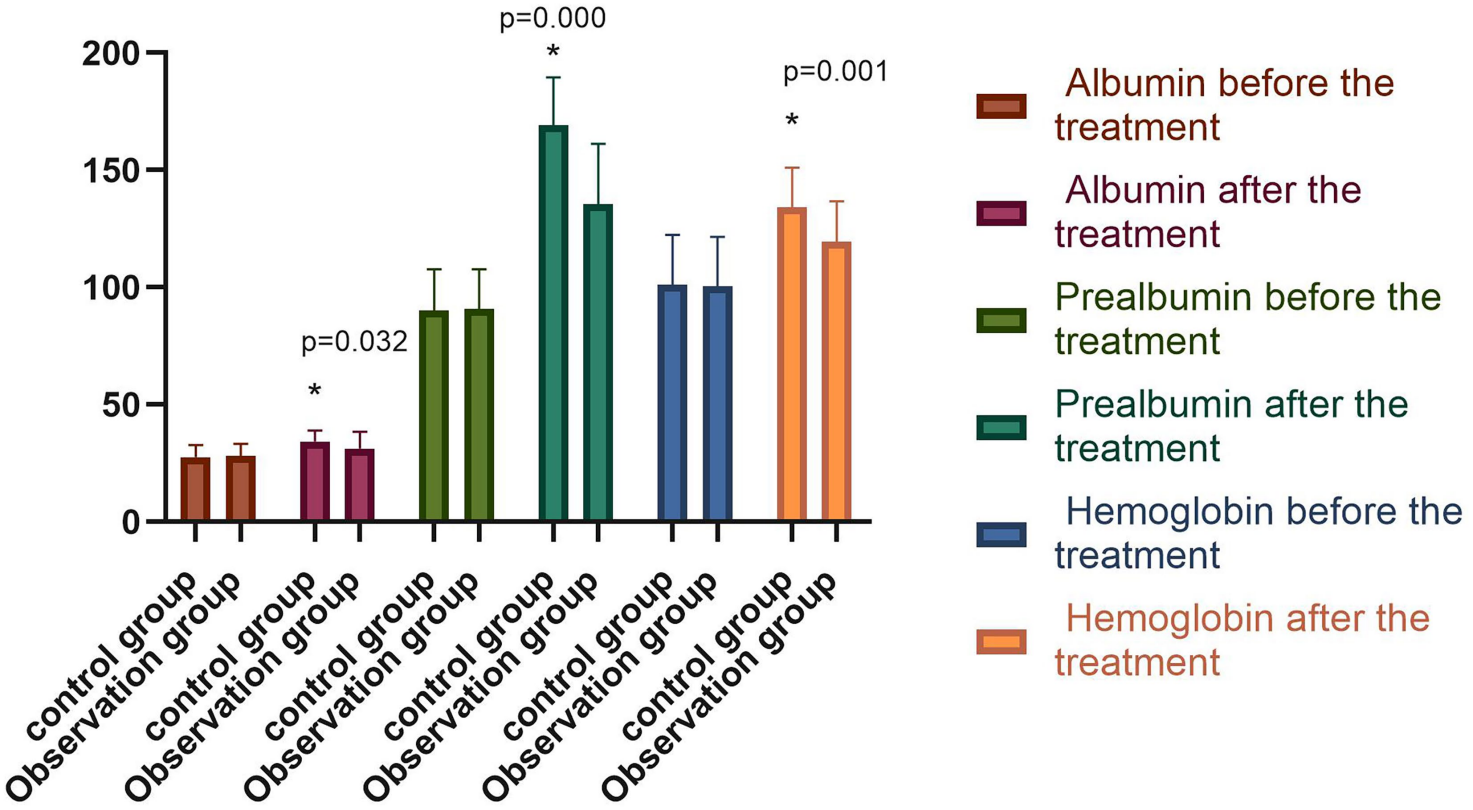
Comparison of serum nutritional indexes of both groups before and after the treatment (^−^*x* ± *s*). Compared with the group before the treatment, *p < 0.05.

**Figure 6:**
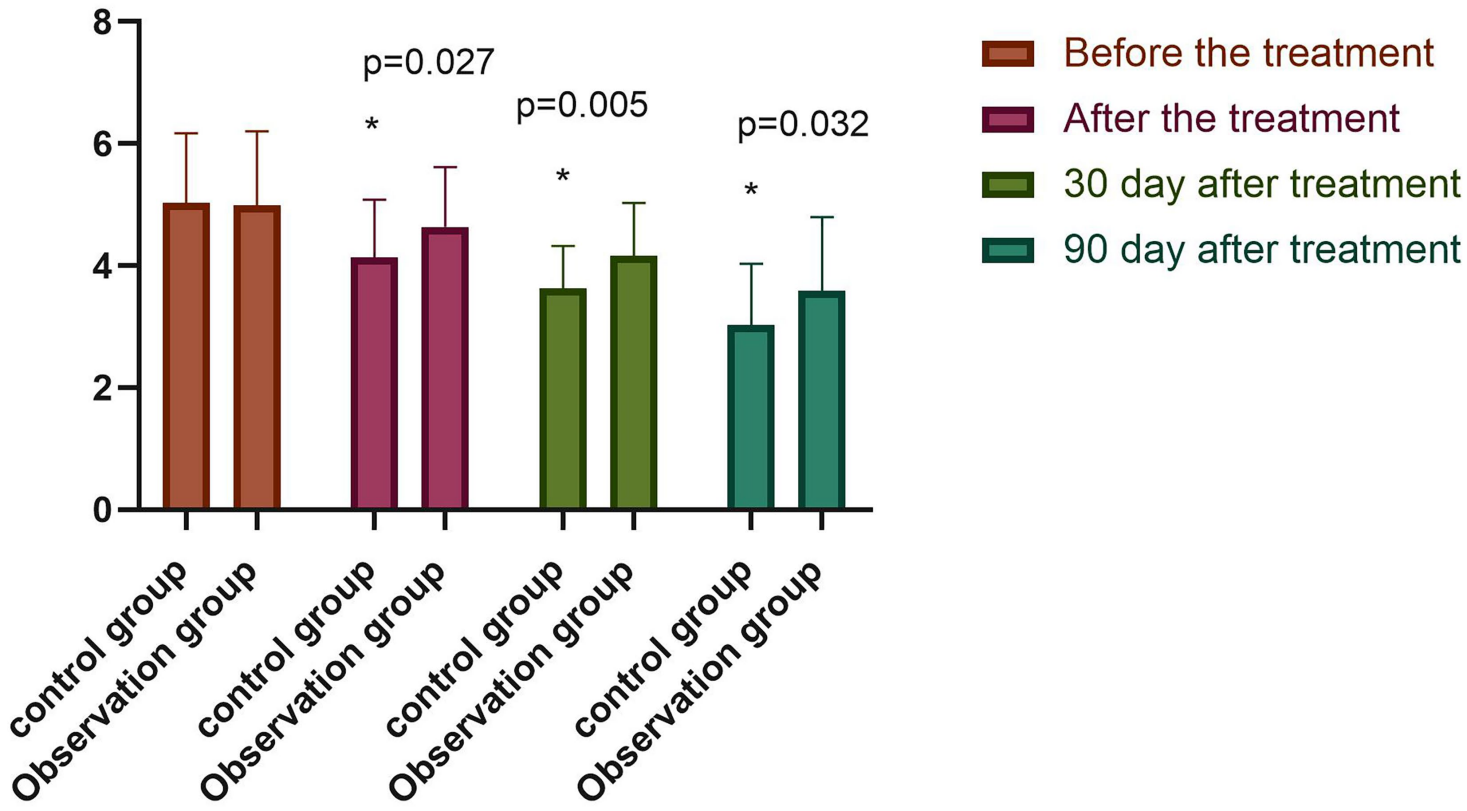
Comparison of NRS2002 scores between both groups (^−^*x* ± *s*). Compared with the group before the treatment, *p < 0.05.

The differences in serum albumin, prealbumin, and hemoglobin levels between the two groups before the treatment were not statistically significant (*p* > 0.05). However, following the treatment, the above serum nutritional indices were significantly higher in both groups compared with those before the treatment (*p* < 0.05), with the indices of the observation group significantly higher than those of the control group (*p* < 0.05), as shown in Figure 5. The difference between the NRS-2002 scores of the two groups before the treatment was not statistically significant (*p* > 0.05), with the scores of both groups greater than three points, meaning nutritional support should be provided. Following the treatment, the NRS-2002 score of the observation group was significantly lower than that before the treatment (*p* < 0.05), while the score of the control group was lower than that before the treatment, albeit the difference was not statistically significant (*p* > 0.05). Overall, the NRS-2002 score of the observation group was significantly lower than that of the control group (*p* < 0.05). On the 30th day of the follow-up, the NRS-2002 scores of both groups were significantly lower (*p* < 0.05) following the treatment, with the scores of the observation group significantly lower (*p* < 0.05) than those of the control group. On the 90th day of the follow-up, the NRS-2002 scores in both groups were significantly lower (*p* < 0.05) than those before and after the treatment, and on the 30th day of the follow-up, where the scores in the observation group were significantly lower (*p* < 0.05) than those in the control group, as shown in Figure 6.

## 7. Discussion

In recent years, based on the theory of the MGB axis, the research on the notion of treating the gut from the brain has been increasing, with positive therapeutic effects observed; however, there exist comparatively few clinical studies related to rTMS. It has been found that rTMS can rapidly induce the activation of the dopaminergic reward system (the corpus striatum, the ventral tegmental area, the nucleus accumbens) and promote the release of dopamine through high-frequency stimulation of the prefrontal cortex. At the same time, multiple treatments with rTMS may induce modulation of the norepinephrine transporter protein, leading to changes in the norepinephrine levels throughout the body, which may have a beneficial effect on the composition of the intestinal microbiota (13).

The PVS patients tend to lose their cognitive functioning, have no swallowing ability, and can be in a coma for a long time. They have a high catabolic and metabolic state and negative nitrogen balance. Malnutrition may lead to poor immunity and complications in patients, such as infections, pleural effusion, and pressure sores, making it extremely difficult for them to survive. Most patients in a PVS suffer from an intestinal resting state due to long-term total parenteral nutrition, which leads to a decrease in intestinal absorption capacity and an imbalance in the ratio of the intestinal flora, thus, further aggravating the damage to the intestinal mucosal barrier. Appropriate nutritional support can help patients in a PVS improve their negative nitrogen balance. This not only potentially provides them with appropriate nutrition but also protects the integrity and functioning of the intestinal mucosal barrier, prevents the migration of bacteria and toxins, and reduces the incidence of various intestinal and gastrointestinal complications. Therefore, this enhances the patient’s body’s immune functioning and the foundation of their rehabilitation. Malnutrition is a risk factor for infection in PVS patients and an independent risk factor for poor prognosis. In fact, the mortality risk among PVS patients with a pulmonary infection is around 2.975× higher than among those without such an infection, while the mortality risk among those with a poor nutritional status is around 3.352× higher than among those with a good one (14). Therefore, providing patients with timely nutritional support is important to promote their prognosis and prolong their survival.

It has been reported that PVS patients who received enteral nutrition support exhibited a significant improvement in nutritional status, a significant reduction in the incidence of complications, and an increase in survival rate. It has also been demonstrated in a related study that the nutritional indices of PVS patients, including in terms of blood glucose and total serum protein, were significantly improved following 2 weeks of enteral nutrition support compared with before the treatment (15). The results of this study indicated that the serum albumin, prealbumin, and hemoglobin levels were significantly higher in both groups following the treatment and that the observation group had significantly higher serum nutritional indices than the control group.

It has also been demonstrated that the NRS-2002 system can be applied to screen for patients at nutritional risk and that implementing nutritional support for patients at nutritional risk can help improve the clinical outcomes in terms of, for example, shorter hospital stays, lower incidence rates of complications, and fewer readmissions (16). In the present study, the NRS-2002 scores of both groups before the treatment were > 3, indicating that both groups of patients were at nutritional risk. Following the treatment, the scores of the observation group and the control group decreased compared with those before the treatment, while those of the observation group decreased more significantly. On the 30th and 90th days of the follow-up, the scores of both groups were significantly lower than those before the treatment, with the score of the observation group significantly lower than that of the control group. The overall indication was that the nutritional status of PVS patients could be significantly improved by using high-frequency rTMS combined with enteral nutrition therapy.

In recent years, many clinical studies on the bidirectional regulation between the brain and the gut have focused on treatment that promotes consciousness. In the present study, based on the stimulation of the prefrontal cortex *via* high-frequency rTMS, the MGB-axis theory was used to regulate the gut microorganisms, which, in turn, had a positive regulatory effect on the nutritional state of the patients. In fact, the method proposed in this study is easy to use, highly reproducible, and allows for easy control of the variables, meaning it provides some reference value for the clinical development of clinical treatment plans for patients in a PVS.

However, the study includes a number of shortcomings, mainly in terms of the following two aspects. First, the study did not include the patients’ underlying medication uses as a variable. Some studies have demonstrated that the prevention rate of nutritional risk in patients taking more than three prescription drugs per day is 1.782× higher than that in patients taking fewer than this number of prescription drugs, with the prevalence rate of nutritional risk in diabetic patients found to be 1.659× higher than that in non-diabetic patients, while the prevalence rate of nutritional risk increases by 1.113× for every one-year increase in age (17). As such, it is not known whether the patients’ underlying medication uses would have been an influencing factor in the current study, which may have led to some bias in the results. Second, brain function observation and evaluation indices were not included. For example, the fractional amplitude of low-frequency fluctuation, regional homogeneity, ectocinerea, and white matter volume were not compared based on the resting-state functional magnetic resonance imaging technique. These indices could be used to analyze the difference in brain functioning before and after the two treatments, as well as to measure the brain-derived neurotrophic factor in the serum and compare the PVS scale/coma recovery scale-revised scores before and after the test. Therefore, further study will be conducted in which the above observation and evaluation indices will be introduced in an attempt to explore further and explain whether high-frequency rTMS can improve the brain functioning and the consciousness state of PVS patients through the MGB, thus providing a more effective treatment plan for the consciousness-promoting treatment in PVS patients.

## 8. Conclusion

This paper presented a preliminary study on whether the rTMS technique can modulate the nutritional status of PVS patients through regulating the intestinal flora and the metabolites, with the correlation between them also investigated. The results indicated that the rTMS technique might improve the nutritional status of PVS patients by regulating the structure of the intestinal flora and affecting the level of SCFAs through the MGB axis.

## Data availability statement

The datasets presented in this study can be found in online repositories. The names of the repository/repositories and accession number (s) can be found below: BioProject accession number: PRJNA868950.

## Ethics statement

The studies involving human participants were reviewed and approved by the Fourth Clinical Medical College of Guangzhou University of Chinese Medicine Ethics Committee. The patients/participants provided their written informed consent to participate in this study.

## Author contributions

X-WL and Y-LW conception and design of the research. TP and QW acquisition of data. PX and J-MC analysis and interpretation of the data. D-NW statistical analysis. K-XZ obtaining financing. X-WL and N-NZ writing of the manuscript. X-WL and H-BY critical revision of the manuscript for intellectual content. All authors contributed to the article and approved the submitted version.

## Funding

This work was supported by the special fund for scientific and technological innovation and industrial development of Shenzhen Dapeng New district (YL202001-17).

## Conflict of interest

The authors declare that the research was conducted in the absence of any commercial or financial relationships that could be construed as a potential conflict of interest.

## Publisher’s note

All claims expressed in this article are solely those of the authors and do not necessarily represent those of their affiliated organizations, or those of the publisher, the editors and the reviewers. Any product that may be evaluated in this article, or claim that may be made by its manufacturer, is not guaranteed or endorsed by the publisher.

## Abbreviations

SCFAs, short-chain fatty acids
NRS2002, Nutrition risk screening 2002
MGB, Microbiota-Gut-Brain
RDPFC, right dorsolateral prefrontal cortex
HPLC-MS, high performance liquid chromatography-mass spectrometry
ESPEN, European Society for Parenteral Enteral Nutrition
NET, norepinephrine transporter protein
fALFF, fractional amplitude of low frequency fluctuation
ReHo, regional homogeneity
rs-fMRI, resting-state functional magnetic resonance imaging
BDNF, brain derived neurotrophic factor
CRS-R, scale/coma recovery scale-revised
ANS, autonomic nervous system
NET, norepinephrine transporters
IBT, immunoblotting test
